# The Hygienic Status of Different Forage Types for Horses—A Retrospective Study on Influencing Factors and Associations with Anamnestic Reports

**DOI:** 10.3390/vetsci9050226

**Published:** 2022-05-06

**Authors:** Sandra Intemann, Bernd Reckels, Dana Schubert, Petra Wolf, Josef Kamphues, Christian Visscher

**Affiliations:** 1 Institute for Animal Nutrition, University of Veterinary Medicine Hannover, Foundation, Bischofsholer Damm 15, 30173 Hanover, Germany; sandra.intemann@tiho-hannover.de (S.I.); dana.carina.schubert@tiho-hannover.de (D.S.); josef.kamphues@tiho-hannover.de (J.K.); christian.visscher@tiho-hannover.de (C.V.); 2 Institute for Nutrition Physiology and Animal Nutrition, Faculty of Agricultural and Environmental Sciences, University of Rostock, Justus-von-Liebig-Weg-6, 18059 Rostock, Germany; petra.wolf@uni-rostock.de

**Keywords:** forage, feed hygiene, horse feeding, animal health, microbiology

## Abstract

The hygienic quality of forage for horses is discussed as a potential health hazard, especially regarding respiratory diseases, colic, and hepatopathies. Therefore, the purpose of this study was to investigate the possible relations between microbiological counts, as well as endotoxin levels and disease symptoms. Data from microbiological examination reports were analyzed retrospectively, including the results of sensory examination, microbiological counts, and lipopolysaccharide contents. Sensory analysis gave an indication of deficiencies in microbiological analysis, but both methods did not give consistently equivalent results regarding the hygienic status of forage. The strongest agreements between sensory and microbiological findings were demonstrated in haylage regarding mold contamination. The influences of dry matter content on microbiological quality could be shown in haylage and hay, whereas this did not apply to straw. Deviations regarding molds and the detection of *Aspergillus* species occurred, especially in haylage, with values above 70% DM detected (39.6%, *p*=0.0021 and 47.2%, *p* = 0.0393). *Aspergillus* was detected more frequently, and average counts were higher in samples that were suspected to induce coughing in horses (*p* = 0.0118 and *p* = 0.0313, respectively). The results of the present study emphasize the importance of feed hygiene for equine respiratory health and the need for the microbiological examination of feedstuffs, since sensory analysis cannot provide an error-free prediction of microbial counts.

## 1. Introduction

Horses are adapted to high-fiber feed (forage), since fiber is digested due to fermentation and hydrolyzation by symbiotic microbes, especially in the hindgut [1]. In European horse feeding, hay is traditionally used as a storable forage [2,3,4]. However, hay is being replaced more and more by haylage in countries such as Finland [5], Sweden [6], and Switzerland [7]. The main reason given for this development is the shorter wilting time in haylage preparation [7]. Straw is commonly used as a bedding material [8], and it can be used as an alternative to grass forage for horses with low energy requirements [9], to a maximum level of 50 percent of the forage diet [10].

Regarding the assessment of the hygienic quality of feedstuffs, sensory and microbiological methods are used. In sensory analysis, the visual, olfactory, and haptic appearance of feedstuffs is investigated [11]. Microbiological analysis can be performed by cultivation or biochemical methods to determine the load of aerobic bacteria, yeasts, and molds. Animate factors such as storage pests and microorganisms (bacteria, molds, and yeasts) are major factors in the spoiling process of feedstuffs [12]. It has already been shown that sensory examination of the feed usually provides indications of high-grade microbial loads [13,14].

As a result of excessive microbial growth, the products of microorganisms, such as mycotoxins, or of cell wall components, such as lipopolysaccharides (LPS), can accumulate in the feed and affect animal health [15].

Measures to prevent feed spoilage, and thus to ensure sufficient feed safety are defined as “feed hygiene” in European feed legislation [16]. For the preservation of hay and straw, dry matter (DM) contents of at least 86% are required [16], whereas haylage must consist of 50–70% DM, and it has to be stored under anaerobic conditions.

Besides its effects on feedstuffs, such as nutrient losses and reduced acceptance by the animal [17], potential health hazards emanating from microbiologically contaminated feedstuffs are discussed. Regarding the effects on horses, particular emphasis lays on chronic respiratory diseases and gastrointestinal disorders [12]. Furthermore, liver disorders are common in clinical practice [18], and can derive from toxic substances [19] such as mycotoxins [17].

Chronic, non-infectious respiratory diseases occur with an estimated prevalence of 10–20% in Northern Hemisphere horse populations [20]. In Switzerland, the incidence was estimated to be as high as 50% of the horse population by a randomized study [21]. Clinical presentation and pathogenesis are comparable to asthma in humans, as it is a recurrent obstruction of the airways due to bronchial hyperreactivity to environmental agents [22]. Affected horses show varying degrees of respiratory distress, as well as poor performance and chronic coughing. The terms “heaves” and “Recurrent Airway Obstruction” (RAO) are used synonymously, whereas “Inflammatory Airway Disease” (IAD) specifies a form that is characterized by a milder clinical manifestation, yet with potential deterioration [20]. In more recent publications, it is proposed that the term “Equine Asthma” (EA) be established, since the term RAO is considered to misrepresent the anatomical location and the etiopathogenesis of the disease [23]. Evidence that aeroallergens, such as mold spores and LPS are etiological components of the disease is now largely undisputed [24,25,26,27]. Exposure to endotoxins, forage mites, and mold spores originating from dust from roughage, and subsequent inhalation has been experimentally shown to induce symptoms of EA [24]. Hay is considered to be particularly problematic, which is why the use of haylage as forage for EA-affected horses is suggested [28,29]. It is also recommended that straw is replaced with other bedding material such as wood shavings [30].

Concerning gastrointestinal disorders, colic is one of the most common causes of death in horses [31,32]. It is not defined as a disease, but as a generic form of abdominal pain [33]. The pathogenesis is variable, and it can originate in different parts of the intestinal tract [34,35]. For example, obturation or impaction of the intestinal tube can lead to passage disorders, causing abdominal pain. Dysbiosis may also occur and lead to excessive gas production (tympany) [36]. Overall, the etiopathogenesis of colic is described as being multifactorial, with different aspects of feeding practice also being considered as triggering factors. This has already been shown in the literature for abrupt feed changes [37,38,39], the intake of large amounts of concentrated feed [32,40], restrictive water supply [39], or the feeding of hard-to-digest forage types such as hay of inferior quality [37]. However, the intake of feedstuffs with deficiencies in hygienic quality is also repeatedly discussed as a cause of gastrointestinal dysfunctions [12,41,42,43]. Abnormally high microbial loads of the feed are seen to potentially irritate the enteric nervous system, and therefore disturb intestinal motor function [44]. A significant correlation between bacteria and yeasts in hay and colic was found in an Austrian study [43]. Moreover, an excessive intake of yeasts can lead to gas formation in the stomach [35,44,45]. Furthermore, in the case study by Kamphues and Boehm [46], five of six horses suffered from colic after being fed oats contaminated with high mold counts. Meyer [42] reported that elevated mold contents of above 10^4^ colony-forming units (cfu) per gram were frequently detected in feedstuffs that caused colic symptoms. However, further findings on statistically significant correlations between increased microbial loads in the feed and disorders of the gastrointestinal tract are desirable.

Studies in dairy cows have shown that increased mold and bacterial counts in the feed leads to a time-delayed increase in the serum levels of liver enzymes, such as aspartate aminotransferase (ASAT) and glutamate dehydrogenase (GLDH) [47]. Elevated GLDH levels indicate hepatocellular damage, whereas ASAT is a less specific parameter indicating liver cell- as well as cardiac or skeletal muscle cell destruction [19]. Hepatogenic photosensitization can be explained by mycotoxin contamination of the feed [48]. A correlation between mold counts and mycotoxin contamination has already been demonstrated in the past, for fumonisins, as well as for zearalenone [47].

In this respect, there are indications that microbial contamination may not only trigger equine chronic respiratory disease, but also gastrointestinal and liver diseases in horses, which this study sets out to investigate.

## 2. Materials and Methods

The data for our study were derived from archived diagnostic data from the Institute of Animal Nutrition, University of Veterinary Medicine Hannover, Foundation, Hanover, Germany. Data were available for samples submitted for microbiological examination in the years 1993–1999 (period 1), as well as 2010–2016 (period 2). Therefore, the present study is a retrospective evaluation of secondary data that were not originally collected for this purpose.

### 2.1. Data Collection

Data were derived from the reports of the microbiological analysis of forage submitted by horse owners or veterinary practitioners to investigate the hygienic quality of the material. The information given in the reports included the date of analysis, the type of forage, the anamnestic report, and the dry matter content, as well as results from sensory and microbiological examinations, and the determination of LPS contents. Reports on different types of forage were available for the purpose of this study. Feedstuffs other than hay, haylage, and straw were excluded from the investigation, due to low case numbers. The preliminary report had been requested from the sender when registering the sample, in order to gather information on the reason for its submission, such as the occurrence of disease symptoms that were observed by the sender in horses after ingesting the material. The results of the microbiological analysis contained counts of aerobic bacteria, molds, and yeasts, as well as the germ and mold species that were detected.

### 2.2. Examination Methods

The sensory hygienic status of the forage samples was determined at the Institute for Animal Nutrition in a standardized manner following the procedure described by Kamphues et al. [8]. The feed was checked for deviations in smell, color, texture, and visible impurities (Table 1). Impurities were detected with the naked eye or by using loupe view.

DM contents were determined regularly in submitted samples. For this purpose, the feed samples were dried at 103 °C until weight constancy was achieved [49].

The microbial contents of the forage samples were determined by cultivation at the Institute for Microbiology, University of Veterinary Medicine Hannover, Foundation. The sample material was first diluted with peptone water and then shaken. Until November 2012, 2.5 g sample material was diluted with 247.5 mL peptone water, then 20 g samples and 380 mL of nutrient solution were used. Throughout the investigation period, the dilution was spread directly and in dilution series of 10^−4^ to 10^−8^ (aerobic bacteria) and 10^−2^ to 10^−5^ (yeasts and molds) in intervals of 10. As culture media for bacteriology, blood agar and Gassner plates were used. Since November 2012, Gassner plates have only been used if the sender requests that counts of *Enterobacteriaceae* should be determined. Mycological examination was performed on a fungal culture medium with antibiotic additives produced at the Institute of Microbiology, University of Veterinary Medicine Hannover, Foundation (“Hamburger Testagar”), throughout the investigation period. The samples were then incubated for two days at 37 °C (aerobic bacteria), and for five to seven days at 30 °C (yeasts and molds). Bacterial count plates in the dilution series harboring approximately 50 colonies were counted to determine the cfu per gram sample. If growth could only be determined on the slide that had not been diluted, the bacterial count was designated as being “below 10^5^” for bacteria, and “below 10^3^” for yeasts and molds. In cases where the bacterial count was above the detection limit, the predominant germ species were identified using biochemical methods. In mycology, separate counts were given for the different genera. Yeasts were not differentiated.

The LPS content was only determined in the first investigation period by means of the limulus amoebocyte lysate test (LAL test) [50]. For this purpose, 10 grams of the feed sample was incubated in distilled water at 80 °C for one hour. Dilution series were prepared from the supernatant obtained after centrifugation. These were incubated with the test solution on titer plates, to determine the concentration in µg/g of the original substance at which gel formation no longer occurred.

### 2.3. Assessment of Examination Results

Due to the totality of the deficiencies identified in sensory examination, the sensory hygienic status of hay, haylage, and straw was rated. For this purpose, the hygienic status was quantified and scored according to Kamphues et al. [8] (Table 1).

Deductions were then added up and the sensory score was determined on the basis of the total points (Table 2).

Regarding the DM content, the values were considered to be too low when they undercut 86% in hay and straw samples. Haylage should consist of 50–70% DM [12], which is why the values undercutting or exceeding this recommendation were assessed as being deviations.

The determined plate counts were compared with the current reference values stated by the Association of German Agricultural Investigation and Research Institutions (VDLUFA) [51]. The VDLUFA orientation values for hay, silage, and straw are shown in Table 3.

The orientation values represent the upper limits of the usual microbial content of various feedstuffs. They were developed for different types of feed. Particular values are stated for the counts of spoilage and product-typical bacteria and molds, while for yeasts, a total count is given. Since no orientation values have been established for haylage, the values for silage were applied.

The level of the determined LPS value was classified as being “generally harmless” (<20 µg/g), “elevated” (20–50 µg/g), and “excessive” (>50 µg/g), in accordance with Kamphues [50]. Excessive levels of lipopolysaccharides were assessed as deficiencies.

### 2.4. Statistical Analyses

Descriptive and analytical statistics were performed using the SAS^®^ Enterprise Guide 7.1^®^ for Windows. Chi-squared tests and Fischer’s exact test were performed to test the association of deviating DM contents and microbiological deviations. For this purpose, deviation frequencies regarding bacteria, molds, and yeasts of hay and straw samples with DM contents below 86% were compared with the frequencies of samples with DM contents of at least 86%. Regarding haylage, proportional frequencies of inadequate microbial counts were compared between the groups (“DM below 50%”, “DM between 50 and 70%”, and “above 70%”). Table analysis was also used, to verify whether the occurrence of health disorders such as colic, coughing, and elevated liver enzymes were associated with deviating microbial counts. For these analyses, samples that had been submitted for routine examination were considered as a control group. The agreement between sensory and microbiological analyses considering the hygienic quality assessment of forage types was evaluated by calculating Cohen’s Kappa (ĸ).

## 3. Results

**Figure 1:**
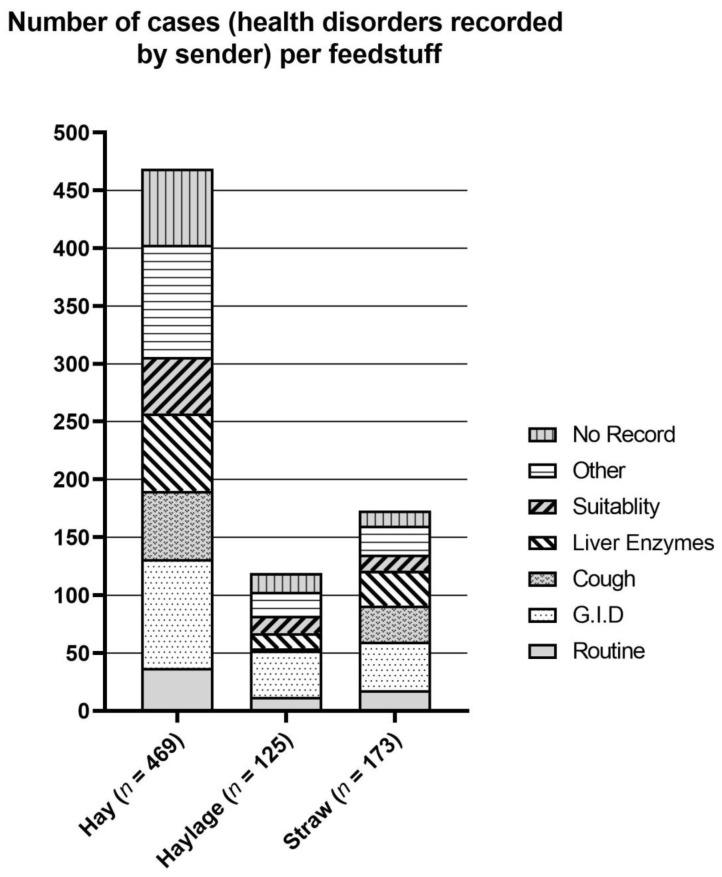
Anamnestic reports given for samples of hay, haylage, and straw submitted for microbiological examination. Other (*n* = 149, all types of roughage): Allergic symptoms (30), refusal of feed (27), laminitis (15), poor performance (14), sudden death (12), cachexia (11), skin disease (4), swelling (legs) (4), intoxication symptoms (3), official control (3), abortion (3), fungal infection (3), swelling (head) (3), sterility (3), nervous disorders (2), lameness (2), sudden collapse (2), bale inflammation (1), salivation (1), cystitis (1), alteration of blood counts (1), botulism (1), Equine Cushing’s disease (1), fever (1), polyuria (1). G.I.D. = gastrointestinal disorder (*n* = 195): colic (169), diarrhea (16), or watery stools (10).

During the investigation period, a total of 838 forage samples were microbiologically tested. Of these, 365 samples were examined in the year range 1993–1999, and 473 in the year range 2010–2016. The distribution of the case history for the three types of roughage considered is shown in Figure 1.

Proportionally, hay was the feed type most frequently examined (55.6% of forage types). Hay and haylage samples were submitted more frequently in the second period than in the first (*p* = 0.0009).

The proportionate frequencies of the reasons for examination were, in descending order, the occurrence of gastrointestinal disorders after feeding (colic/diarrhea/watery stools; 23.3%), elevated liver enzymes in horses fed the material (13.5%), unknown reasons (12%), the occurrence of coughing after feeding (11.7%), verifying suitability as horse feed (10.5%) routine examinations (8.5%), and other reasons (each less than 4%; total 20.3%).

### 3.1. Proportional Frequency of Deficiencies

An overview of the examination results regarding the hygienic status of forage samples is given in Table 4.

#### 3.1.1. Dry Matter Content

Overall, in 37% of the hay samples and 22% of the straw samples, the DM content was below 86% (see Table 4). The DM content of haylage samples was inadequate in 62.2% of the cases. A DM content below 50% was found in 26% of the haylage samples, while 27% showed values of 55–70%, and 47% showed values above 70% DM. The average DM content amounts were 65.3%, with an average pH value of 6.3. All but one of the haylage samples had values below the 86% DM content recommended for hay.

#### 3.1.2. Sensory Control

The sensory analysis revealed that the proportion of deviations was the highest for straw (*p* < 0.0001), whereas approximately 44% of hay and haylage samples were inadequate (Table 4). Frequently mentioned deviations in hay and straw samples included a musty odor (33.6% and 37.6%, respectively), a clammy texture (30.4 and 19.4%, respectively), a grayish color (22.2 and 63.5%, respectively), and deposits (15.3 and 45.3%, respectively). The described smell for haylage was yeasty–alcoholic in 26.6% of the cases and moldy–musty in 29.8% of the cases, whereas a grayish color was observed in 25% of the samples. The texture was found to be inadequate in 8.1% of the cases, and deposits were detected in 24.2% of haylage samples.

#### 3.1.3. Microbiological Quality of Feed Types

When considering the counts of aerobic bacteria, molds, and yeasts, deficiencies were determined in 38% of the examined feedstuffs (see Table 4). The frequency of high contamination with molds did not differ significantly between feedstuffs, whereas exceedingly high counts of yeasts and bacteria occurred more frequently in haylage samples than in hay and straw samples (*p* < 0.0001). For example, 4.6% of hay samples were found to have yeast counts above 1.5 × 10^5^ per gram sample, while this share was 28.6% for haylage. LPS contents of above 50 µg/g sample were determined most frequently in straw (73.0%).

When haylage samples were evaluated according to the orientation values for hay, the frequencies of inadequate aerobic germ counts decreased from 55.9% to 20.8% (*p* < 0.0001). Mold counts were assessed as being inadequate less frequently when using orientation values for hay as well (*p* = 0.0460), while the frequency of deviating yeast counts did not change significantly (Table 4).

Between the two periods, there were no significant changes in the frequency of the microbiological deviations in hay, haylage, and straw.

In cases where the total aerobic germ counts were given, the predominant species were indicated in the report. The proportional cultivation frequencies of bacterial species classified in groups according to VDLUFA [51] are shown in Table 5.

In straw samples, 42.9% of the detected bacteria were classified as being feed-typical, and 56.8% as spoilage bacteria, whereas in the hay samples, 20.7% feed-typical and 78.3% spoilage bacteria were detected (Table 5). The situation was similar for haylage (18.9% feed-typical and 75.3% spoilage bacteria). Only in six of the 125 haylage samples could *Lactobacillus* be cultivated as the predominant germ species.

### 3.2. Relationships between Parameters

#### 3.2.1. Dry Matter Content and Microbiological Quality

Table 6 shows the proportional frequency of exceeding counts of bacteria, molds, and yeasts in feedstuffs with normal and low DM contents.

A low DM content of hay was associated with an excessive yeast population (*p* = 0.0018; Table 6). Elevated mold levels occurred less frequently in hay and straw, with low DM contents than in samples with contents above 86%, but the difference was not significant. Excessive counts of aerobic bacteria were more frequently observed in DM contents <86% (15.2% vs. 9.8%), but again, no significant association was found (*p* = 0.1393).

Haylage with excessively high and excessively low DM contents differed in terms of aerobic germ counts, since the proportion of inadequate samples was greater in low-DM samples (*p* = 0.0393). The proportion of haylage samples with an increased mold count was greater in samples with a DM content above 70% than in samples with DM values between 50 and 70% (*p* = 0.0021). *Aspergillus* species were detected more frequently in high-DM samples (*p* = 0.0298, Table 6) than in samples with an optimal DM content. Samples with DM contents below 50% did not differ significantly from those with optimal DM contents regarding microbiological quality. While 11.6% of the haylage samples with optimal values showed excessive mold growth, 27.8 and 39.6% of the haylage samples that were too low and too high in DM content, respectively, showed excessive mold contamination.

#### 3.2.2. Association of Sensory and Microbiological Deviations

The agreement of sensory and microbiological analyses was calculated to assess whether exceedingly high microbial counts could be predicted sensorily (Table 7 and Table 8). This was performed for roughage samples in total, as well as for the different feed types (hay, haylage, and straw). Table 7 shows the results of this investigation regarding aerobic bacteria.

Agreements with exceeding germ counts could only be shown for the sensory parameters in the analyses of haylage (Table 7). There, a fairly good degree of agreement was observed between abnormal odor, as well as abnormal color and deviating germ counts (ĸ = 0.3341 and 0.2167, respectively). The combination of those parameters also showed a fairly good agreement with the excessive growth of aerobic bacteria (ĸ = 0.2073).

A fair agreement was shown for the excessive contents of cell wall components of gram-negative bacteria (LPS) and a deviating sensory score in roughages in general (ĸ = 0.3324), as well as hay in particular (ĸ = 0.3530). Deposits have shown to agree with deviating LPS content (ĸ = 0.2091) in hay, whereas this applied to roughage and straw only in combination with the abnormal color of the samples (ĸ = 0.2444 and 0.2732). No calculations were made for haylages, since LPS content was only determined in 10 cases.

The results of calculations performed for molds and yeasts exceeding orientation values are shown in Table 8.

Regarding the inadequate sensory score, a fair amount of agreement was shown with the deviating mold counts in hay (ĸ = 0.2097), and a moderate amount of agreement with mold counts exceeding the orientation values in haylage (*p* = 0.4347, Table 8). The agreement of abnormal odor (moldy/musty) and the deviations of mold counts could be shown in all feedstuffs except straw. In haylage, a moderate agreement of abnormal color (ĸ = 0.4018), as well as deposits (ĸ = 0.4062) and excessive mold counts were shown. In contrast to this, the agreement of deviations regarding yeasts could only be observed with deposits in haylage.

A moderate amount of agreement was observed regarding the combination of abnormal odor and deposits, and the excessive growth of molds (ĸ = 0.4159). The deviations in color and deposits, as well as in odor and deposits were in fairly good agreement, with the mold counts exceeding the acceptable values (ĸ = 0.3770 and ĸ = 0.3952).

#### 3.2.3. Relations between Reported Disease Symptoms and Microbiological Deviations

Coughing after feeding, as reported by the sender of the material, did not occur significantly more often in forage samples whose plate counts were found to exceed the orientation values for microbiological quality, according to VDLUFA (2017) [51] (Table 9), and neither the detection of storage mites nor LPS-counts above 50 µg per gram sample in hay and straw were associated with the occurrence of coughing.

However, *Aspergillus* species were detected more frequently in roughage that was reported to induce coughing, than in samples that were intended for routine examination (*p* = 0.0118, Table 9). This also applied to hay samples in particular (*p* = 0.0216). Furthermore, the average count of *Aspergillus* was higher in samples where coughing was observed after feeding (*p* = 0.0313); see Table 10.

Neither excessive LPS contents nor the detection of storage mites were associated with the occurrence of coughing (Table 9). No calculations were made for haylage samples that had been submitted to clarify the cause of coughing, as only a total of three samples were examined in this preliminary report.

The same analyses as presented in Table 9 were performed for the occurrence of gastrointestinal disorders and liver enzyme alteration. Deficiencies in the microbiological quality did not occur significantly more frequently in any of the roughage types when gastrointestinal disorders or liver enzyme alteration were observed after feeding.

## 4. Discussion

### 4.1. Sensory Quality

In the present study, regarding haylage and hay, hygienic status was evaluated as being inadequate due to sensory examination in 44.4% and 44.2% of the cases, respectively. In the study by Stickdorn [13], this was the case for 70% of hay and 100% of haylage samples. In that study, however, the case numbers were lower, since 15 straw samples, 81 hay, and 5 haylage samples were evaluated.

In our study, the majority of straw samples (67.1%) showed major or massive deviations in sensory analysis (see Table 4). Stickdorn et al. [13] found moderate and significant deviations in as much as 80% of the straw samples. The same scheme of sensory analysis was used, and the study was based on the diagnostic data of horse feed samples submitted to the Chair of Animal Nutrition and Dietetics in Munich, Germany, between 2004 and 2009. Sander et al. [52], on the other hand, found only 37.3% of straw samples to have major or massive deficiencies in sensory analysis. In their study, 153 straw samples submitted between 2009 and 2014 were sensorily evaluated according to Kamphues et al. [8]. Although there is a partial overlap with the data of our study, Sander et al. [52] also included straw samples of pig and poultry farms, which is why the data basis is ultimately different. This could explain the divergent results.

In general, other studies produced results contradictory to the present study. This leads to the assumption that sensory analysis itself or the evaluation of its findings may be subject to bias.

### 4.2. Microbiological Quality of Different Forage Types

When comparing the microbiological quality of feedstuffs for horses, special attention was paid to differences in the deviation frequency of hay and haylage, since haylage is increasingly being used to replace hay as a feedstuff [7]. Molds of the genus Aspergillus were detected more frequently in hay than in haylage (54.6 vs. 31.7%; *p* < 0.0001). Furthermore, 55% of haylage samples had mold counts below the detection limit, whereas this share was 15.9% for hay samples.

Exceedance in counts of aerobic bacteria, molds, and yeasts were evaluated according to VDLUFA orientation values for hay, straw, and silage, respectively. No significant difference was found between hay, haylage, and straw, regarding the proportional frequencies of germ, mold, or yeasts counts exceeding VDLUFA orientation values. Still, the fact that the VDLUFA has not yet defined the orientation values for evaluating haylage complicates the classification of a microbial count as being “above average” for this feedstuff. Despite limited fermentation [53], haylage is, as with the case for silage, stored anaerobically, and it is recognized as a wrapped forage [3]. Therefore, it was assessed using the orientation values for silage, and not for hay. For future analyses, it would be desirable to have separate values for haylage in order for the hygienic quality to be evaluated more accurately.

Mold contamination and the frequency of *Aspergillus* detection were lower in haylage compared to hay samples (*p* < 0.0001). Vandenput et al. [29] showed that the contents of Aspergillus fumigatus in dust emitted by haylage were lower than in dust fractions of hay, but the difference was not found to be significant. However, since the amount of dust emitted by haylage was lower, they recommended feeding haylage instead of hay to EA-affected horses. This recommendation can be further confirmed by our results.

In terms of yeast load, haylage was more frequently affected by excessive yeast load than hay and straw (28.6 vs. 9.9 and 4.6%, respectively). Although the potential risks of exceeding the recommended yeast load in the feed, resulting in gastric tympany and finally colic in horses, is discussed by Frape [45], to our knowledge, no statistical evidence has been shown for this hypothesis in literature so far. Therefore, the potential risks posed by excessive yeast in haylage cannot be conclusively assigned.

### 4.3. Associations between Dry Matter Content and Microbiological Deviations

For the associations between DM content and microbiological quality, a threshold of 86% stipulated in European legislation was used for hay and straw [16]. In hay samples with values below 86%, yeast counts exceeded orientation values more frequently (*p* = 0.0018), but the same did not apply to germ and mold counts. In other publications, such as the review by Vervuert et al. [1], a water content of less than 15% (>85% DM) is cited as being a reference value for good storage stability. The authors of the present study performed the same calculations when applying a reference value of 85% DM content. This did not lead to any changes in the results.

It has to be considered that cultural methods determine the number of viable microorganisms, and that this amount varies from harvest to storage [54]. Thus, excessive mold proliferation may have taken place earlier during the storage period, whereas at the time of examination, excessive contamination was no longer detectable by cultivation.

In haylages, mold counts were assessed as being elevated more frequently in samples with DM contents above 70% than in samples consisting of 50 to 70% DM (*p* = 0.0021). The same applied to the detection frequency of fungi of the genus *Aspergillus* (*p* = 0.0298). Inadequacies regarding aerobic bacteria were observed more frequently in samples that consisted of less than 50% DM than in samples with contents above 70% (*p* = 0.0393). Thus, it can be stated that the risk of hygienic deficiencies is lowest for haylages that consist of 50–70% DM. Fermentation in haylages is known to be restricted due to high DM contents, and thus, anaerobic storage is required to prevent mold growth [3]. It has been shown before that the DM content of silages is correlated with pH and lactic acid concentration in baled grass silage [55]. O’Brien et al. [56] found that the DM content of baled silage had an influence on the composition of fungal species colonizing the bales, but that this was not correlated with the proportion of the bale surface area visibly contaminated by fungi. Müller et al. found that silages with DM contents of 35 and 50% were not significantly different from each other in mold and yeast counts [57]. It can be concluded that although no linear correlation of DM contents and mold counts have been observed in the literature, the exceedance of certain DM contents in haylages increases the risk of mold spoilage. The results of the present study support the recommendation that haylage DM contents should not exceed 70%.

### 4.4. Association of Sensory and Microbiological Deviations

In hay and haylage, abnormalities in odor were in fairly good agreement with increased mold contamination (ĸ = 0.2329 and 0.3667, respectively, Table 8). Other parameters of the sensory examination, such as color and deposits, also provided indications of deficiencies in microbiological quality in these feeds (Table 7 and Table 8). Concerning the sensory analysis of straw, only slight agreements could be shown for yeasts, molds, or bacteria.

The presented results are partially consistent with the results of Wolf et al. [14]. The authors of this previous study indicated a correlation between a dusty character, as well as a moldy smell, and excessive mold contamination of feedstuffs. A yeasty smell was also associated with exceeding yeast plate counts. Additionally, a clammy texture was associated with low DM contents, which are generally associated with increased microbial populations, according to Wolf et al. [14]. In contrast to Wolf’s study, the correlations between sensory perception and microbiology in our study were investigated for roughage only. Furthermore, additional information such as a dusty character was not recorded, following the scheme of Kamphues [8].

Stickdorn et al. [13] investigated the correlation between sensory and microbiological analyses. They found that the sensory score correlated with plate counts of yeasts (*p* < 0.05, *r*^2^ = 0.79) and molds (*p* < 0.001, *r*^2^ = 0.57), but not with the total aerobic bacterial count (*r*^2^ = 0.04). Sensory analysis was also carried out according to the scheme of Kamphues et al. [8], as in the present study. Stickdorn et al. [13] additionally distinguished between no, minor, moderate, and significant deviations, but not between the parameters of the sensory examination, such as odor or color. In this respect, the approaches of both studies were somewhat different, which complicates a direct comparison of the results.

All in all, abnormal odor or color, as well as visible deposits on forage can provide indications of microbial counts that exceed VDLUFA orientation values, especially regarding molds and bacteria. The fact that the storage duration of the samples was not known has to be considered, since cultural methods determine the counts of viable microorganisms, and this number varies along with the storage period.

### 4.5. Association of Microbiological Deviations and Pre-Reported Disease Symptoms

Another aim of this evaluation was to shed light on how often the cause of coughing, gastrointestinal disorders, or elevated liver enzymes in horses could be clarified by microbiological examinations of the feed. It has to be emphasized that, as the data were analyzed retrospectively, no standardized clinical examination of horses could be performed. Thus, the etiology of the symptoms that were observed in horses by the sender after feeding of the material can not be conclusively clarified. The findings of the present study can therefore be seen as indicative, and not as evidentiary regarding the induction of diseases by feed-borne microorganisms.

#### 4.5.1. Coughing

Significant associations could be established between the symptom “coughing” and the presence of *Aspergillus* (*p* = 0.0261). This is consistent with the results of Clarke et al. [24], who experimentally induced coughing through the exposure of horses to endotoxins and forage mites, and mold spores from dust from roughage. McGorum et al. [58] induced pulmonary disease due to the inhalation of extracts of *Aspergillus fumigatus* only in horses already affected by EA, whilst eight horses of the control that were unaffected by EA showed no symptoms after this treatment. In our study, no information was available on the preliminary health status of the horses that were fed the forage. Therefore, we cannot make any statements here about whether the associations that we observed concern healthy horses, or whether they only apply to those animals that were preliminarily affected by EA.

Total mold counts exceeding the orientation values of VDLUFA were not found to relate to the occurrence of coughing after feeding in our study, although Ward et al. [59] found that the prevalence of EA was correlated positively with outdoor mold concentrations.

No connection could be established between the LPS contents of hay, haylage, and straw, and the occurrence of coughing. In contrast to this, Pirie et al. [60] experimentally induced detectable lung dysfunction in EA-affected horses through the inhalation exposure of horses to LPS. Healthy horses were not affected by this treatment. In another study [27], it was shown that endotoxin inhalation plays a role in inflammation and lung dysfunction in horses with EA, and that LPS acts synergistically with other etiological factors such as *Aspergillus fumigatus.*

In the present study, the contents of molds and LPS in the feed itself were used as parameters. The content of inhalable dust with particle sizes below 5 µg was not determined in the feed. In the literature, however, the concentration of inhalable dust emitted by feedstuffs is stated as being an important etiological factor of the disease. The concentration of dust in the air has been shown to influence both the severity [61] and the duration [62] of symptoms of EA. As respirable dust content was not taken into account in our study, no conclusive statement can be made on the triggering of coughing by feedstuffs containing bacterial endotoxins, mites, or exceeding mold counts.

#### 4.5.2. Gastrointestinal Disorder

It could not be shown that deviations in microbiological quality occurred more often in feedstuffs suspected of having caused gastrointestinal disorders. Meyer et al. [42] reported that “[…]after feeding mold-infested hay, disturbances in the digestive and respiratory tracts were almost regularly mentioned […]”. The data included 133 reports of roughage samples, 30 of which were objected regarding mold counts. In 6 of these 30 cases, the samples were submitted due to gastrointestinal disorders, and in one case, due to respiratory disorders. Meyer et al. [42] used an orientation value of 10^4^ colony-forming units per gram of feed for molds. In the present study, the VDLUFA orientation values of 2 × 10^5^ cfu/g of feed-typical and 2 × 10^3^ cfu/g of spoilage molds were used for hay and straw, and a threshold value of 5 × 10^3^ cfu/g of all molds for haylage. Therefore, the study findings of Meyer et al. [42] can be compared with our results only to a limited extent.

Kaya et al. [43] suggested a connection between hay that is excessively colonized with yeasts or bacteria, and the occurrence of colic in horses. They considered a population of more than 10^4^ colony-forming units (cfu) of yeasts, 10^3^ cfu/g of molds, and 10^6^ cfu/g of aerobic bacteria as being inadequate. Similar to Meyer et al. [42], no distinction was made between product-typical and spoilage microorganisms. According to VDLUFA [51], however, values of up to 3 × 10^7^ cfu/g of product-typical bacteria in hay are acceptable. Furthermore, Kaya et al. [43] used a different culture medium and incubation temperature (25 °C) for mycology. The growing conditions for bacteria were different, insofar as bacteria were incubated one day longer at a lower temperature (30 °C). According to Müller et al. [53], the incubation temperature has a significant influence on the type and quantity of fungi detected, as well as on the yeast counts. These circumstances lead to the fact that the comparability of both results must be considered to be limited.

#### 4.5.3. Liver Enzyme Alteration

In our study, no evidence could be found that excessive mold contamination in feedstuffs might affect liver function in horses. Still, Kellerman et al. [48] demonstrated hepatogenic photosensitization in horses after feeds contaminated with mycotoxins were ingested. Furthermore, Anacker [63] indicated a time-delayed increase in the serum levels of ASAT and GLDH in dairy herds after intakes of feed with increased germ and mold contamination. However, in the present study, there was no information given regarding the type of liver enzymes that were conspicuous by elevation in the equine blood serum. Furthermore, no information is available on whether normal values were found in earlier blood samples, and thus whether an increase in these values was observed after the suspected material was fed.

## 5. Conclusions

Comparing the hygienic quality of different forage types, it can be concluded that hay potentially poses greater health risks to horses than haylage, since mold counts exceeding the maximum limit and the detection of *Aspergillus* sp. are more likely to occur in hay samples.

Furthermore, the effect of DM contents on the hygienic quality of forage revealed that haylage should consist of 50–70% DM, since microbial counts exceeding the accepted limit occurred less frequently than in samples with higher or lower values. Again, this is relevant for the feeding of horses suffering from chronic lung diseases, since contamination with molds in general and with *Aspergillus* spp. in particular occurred more frequently in haylage with DM contents above 70%, than in samples with adequate contents.

Regarding the agreement of sensory and microbiological analysis, it was shown that there was no strong agreement between inadequate results in sensory and microbiological analysis. Thus, sensory analysis may indicate an excessive growth of microorganisms, especially of molds and bacteria in haylage. Nonetheless, microbiological examination cannot be dispensed with, due to the findings of the sensory analysis.

The present study did not provide any clear indications on harmful effects on horses caused by counts for bacteria, molds, or yeasts that exceeded the orientation values of VDLUFA. Still, it must be pointed out that the orientation values only reflected the framework of the usual microbial population in the field [64]. These values were determined without taking into account the possible harmful effects of the relevant microorganisms. The threshold values for harmful effects due to excessive colonization with bacteria, molds, or yeasts have not yet been experimentally determined. It must be expected that the reporting of disease symptoms was incomplete or incorrect in some cases, since anamnestic reports were given by persons that were not trained for this purpose, and standardized clinical examination of the horses could not be performed retrospectively.

All in all, new insights could be gained on the effects of high DM contents on the microbiological quality of haylages, and on the limited predictability of microbial counts by sensory examination. Furthermore, it was shown that clarifying disease causes by the examination of feed hygiene was not possible in most cases, which does not exclude the possibility that the feeding of hygienically inadequate forage might promote diseases in horses.

## Figures and Tables

**Table 1:** Evaluation of the hygienic status of hay, straw, and silage by sensory analysis in accordance with Kamphues et al. [8].

Parameter	Hay and Straw		Silage	
	Findings	Points Deducted	Findings	Points Deducted
Odor	Unremarkable	0	Unremarkable	0
			Yeasty nuances	–2
			Distinct yeasty, alcoholic	–4
	Musty nuances	–5	Slightly moldy, musty	–6
	Moldy/putrid	–10	Moldy, rotten, or fecal odor	–10
Texture	Dry	0	Slight to distinct heating	–2 to –4
	Sightly clammy	–2	Slight to distinct loss of structure	–2 to –10
	Clammy, moist	–5	Above average contamination with sand/soil	–2 to –6
Color	Product—typical	0	Unremarkable	0
	Hay: focally gray, whitish	–2	Whitish, grayish, greenish, blackish color deviations/deposits	
	Hay: Diffusely discolored Straw: Dirty grayish/brownish/blackish	–5	Scattered discoloration	–4
	Straw: Whitish gray/red discoloration	–10	Frequent discoloration	–10
Impurities (deposits, visible mold growth/infestation with storage mites)	None	0	Containing waste material, poisonous plants, plant parts altered by disease	–2 to –10
	Medium-grade	–5		
	High-grade	–10		

**Table 2:** Determining sensory score on the basis of points deducted in sensory analysis in accordance with Kamphues et al. [8].

Hygienic Status	Total Points Deducted according to Sensory Analysis		
	Hay	Straw	Silage
Adequate	0	0	0
Minor deficiencies	–1 to –5	–1 to –5	–1 to –5
Major deficiencies	–6 to –10	–6 to –10	–6 to –10
Massive deficiencies	–11 to –40	–11 to –30	–11 to –46

**Table 3:** Orientation values for germ counts of hay, silage, and straw according to VDLUFA [51].

Type of Micro-Organism	Classification	Group No ^1^	Exemplary Species	Orientation Value (cfu/g feed)		
				Hay	Silage	Straw
Aerobic bacteria	“Epiphytic” (product-typical) bacteria	1	*Pantoea agglomerans, Pseudomonas, Enterobacteriaceae*	30 × 10^6^	100 × 10^6^	0.2 × 10^6^
	Spoilage bacteria	2	*Bacillus, Staphylococcus*, *Micrococcus*	2 × 10^6^	2 × 10^6^	0.2 × 10^6^
		3	*Streptomyces*	0.15 × 10^6^	0.15 × 10^6^	0.01 × 10^6^
Molds	“Epiphytic” (product-typical) molds	4	*Aspergillus, Penicillium*, *Scopulariopsis*	200 × 10^3^	200 × 10^3^	5 × 10^3^
	Spoilage molds	5	*Mucorales*	100 × 10^3^	100 × 10^3^	5 × 10^3^
Yeasts		6	all species	5 × 10^3^	5 × 10^3^	5 × 10^3^

^1^ According to classification by VDLUFA (2017) [51].

**Table 4:** Proportional frequency of deviations in the hygienic quality of hay, haylage, and straw samples concerning sensory control (SC), dry matter content (DM), counts of aerobic bacteria, molds, and yeasts, as well as microbiology in total and LPS contents.

Feed Type	*n*	Proportional Frequency (%) of Deviations						
		SC	DM	Aerobic Bacteria ^1^	Molds ^1^	Yeasts ^1^	Micro-Biology (Total) ^1^	LPS >50 µg ^2^
Roughage	767	49.5	38.0	23.5	25.2	10.1	36.9	60.0
Hay	469	44.2	36.9	12.4	25.5	4.6	29.7	52.2
Haylage *	125	44.4	62.2	55.9	27.5	28.6	60.8	40.0
Haylage **	125			20.8	20.8	29.4	53.6	
Straw	173	67.1	22.0	27.0	22.8	9.9	38.3	73.0

^1^ According to VDLUFA (2017) [51]. ^2^ Investigated between 1993 and 1999; assessed according to Kamphues (1986) [50]. * According to VDLUFA orientation values for silage (2017) [51]. ** According to VDLUFA orientation values for hay (2017) [51].

**Table 5:** Proportional detection frequency of aerobic bacteria classified in groups according to VDLUFA [51] in hay, haylage, and straw.

Classification	Group No ^1^	Exemplary Species	Detection Frequency (%) in Forage		
			Hay	Haylage	Straw
“Epiphytic” (product-typical) bacteria	1	*Pantoea*, *Pseudomonas, Enterobacteriaceae*	54.7	54.5	63.7
Spoilage bacteria	2	*Bacillus*, *Staphylococcus, Micrococcus*	38.2	44.5	36.0
	3	*Streptomyces*	7.1	1.0%	0.3

^1^ According to classification by VDLUFA (2017) [51].

**Table 6:** Association of deviating dry matter (DM) contents and plate counts exceeding VDLUFA orientation values [51] in hay, haylage, and straw.

Feedstuff	DM Content	% cfu > n.c. (According to VDLUFA 2017)			*Aspergillus* Cultivation (% positive)
		Aerobic Bacteria	Molds	Yeasts	
Hay	≥86% (*n* = 149)	9.8	26.7	1.9	43.2
	<86% (*n* = 255)	15.2	24.3	9.3	40.3
	Level of significance (*p*-value)				
	≥86% vs. <86%	0.1393	0.6055	0.0018	0.8615
Haylage	<50% (*n* = 18)	77.8	27.8	77.8	11.1
	50–70% (*n* = 45)	58.1	11.6	79.1	25.6
	>70% (*n* = 56)	50.0	39.6	58.5	47.2
	Level of significance (*p*-value)				
	50–70% vs. <50%	0.3425	0.1202	0.7131	0.2081
	50–70% vs. >70%	0.8726	0.0021	0.1900	0.0298
	< 50% vs. > 70%	0.0393	0.3675	0.1422	0.0826
Straw	<86% (*n* = 255)	24.3	19.1	9.4	44.8
	≥86% (*n* = 149)	41.4	24.1	16.0	30.9
	Level of significance (*p*-value)				
	≥86% vs. <86%	0.1151	0.5466	0.3407	0.0722

cfu = colony-forming units per gram sample. n.c. = normal counts according to VDLUFA (2017) [51].

**Table 7:** Agreement of deviations in sensory control (single parameters and combinations of parameters) and exceeding counts of aerobic bacteria, as well as exceeding LPS contents (calculation of Cohen’s Kappa) in different forage types.

Deviations within the Sensory Control	Exceeding Counts of Aerobic Bacteria (% cfu > n.c.) ^1^				Exceeding LPS Content (>50 µg/g) ^2^		
	Forage (*n* = 624)	Hay (*n* = 368)	Haylage (*n* = 118)	Straw (*n* = 138)	Forage (*n* = 145)	Hay (*n* = 86)	Straw (*n* = 49)
Inadequate sensory score	0.1204	0.0963	0.2464	0.0807	0.3324	0.3530	0.1757
Texture	0.0862	0.1463	0.0126	0.1560	0.0174	0.0794	−0.0834
Odor	0.1699	0.1278	0.3341	0.1131	0.1809	0.1891	0.0909
Color	0.2315	0.1464	0.2167	0.1722	0.0281	-	−0.0214
Deposits	0.1192	0.1012	0.0746	−0.0073	0.1363	0.2091	0.0369
Storage mites	−0.0829	−0.0645	0.0268	−0.0187	0.0144	0.0736	−0.0208
Odor + deposits	0.1378	0.1113	0.1264	0.0564	0.1158	0.1108	0.0541
Smell + texture	0.0919	0.1467	0.0197	0.1849	0.0864	0.1131	0.0521
Color + odor	0.2073	0.1167	0.2112	0.1495	0.1694	0.1131	0.1390
Color + deposits	0.1430	0.0474	0.1157	0.0836	0.2444	0.1556	0.2732

cfu = colony-forming units per gram sample. n.c. = normal counts. ^1^ according to VDLUFA (2017) [51]. ^2^ investigated between 1993 and 1999; assessed according to Kamphues (1986) [50]. Cohen’s Kappa (ĸ) < 0 = no agreement; 0 < ĸ < 0.2: slight agreement; 0.21 < ĸ < 0.4 = fair agreement.

**Table 8:** Agreement of deviations in sensory control (single parameters and combinations of parameters) and counts of molds or yeasts exceeding the orientation values (according to VDLUFA [51]; calculation of Cohen’s Kappa) in different forage types.

Deviations in Sensory Control	Mold Counts Exceeding Orientation Values (% cfu > n.c.)				Yeast Counts Exceeding Orientation Values (% cfu > n.c.)			
	Forage (*n* = 704)	Hay (*n* = 426)	Haylage (*n* = 119)	Straw (*n* = 159)	Forage (*n* = 639)	Hay (*n* = 381)	Haylage (*n* = 119)	Straw (*n* = 139)
Inadequate sensory score	0.2164	0.2097	0.4347	0.1167	0.0656	0.0364	0.1713	0.0625
Texture	0.1279	0.1588	0.0121	0.1296	0.0499	0.0661	0.0075	0.1387
Odor	0.2091	0.2329	0.3667	0.0310	0.0614	0.0589	0.0364	0.0716
Color	0.1789	0.1205	0.4018	0.1633	0.1381	0.0962	0.1752	0.0477
Deposits	0.1691	0.1295	0.4062	0.0997	0.1528	0.0589	0.2462	0.0491
Storage mites	0.0574	0.0686	0.0853	–0.0068	–0.0739	–0.0428	0.0246	–0.0560
Odor + deposits	0.1290	0.0897	0.3770	0.0477	0.0972	0.0673	0.1189	0.0099
Smell + texture	0.1597	0.1815	0.0749	0.1670	0.0773	0.1076	0.0156	0.1813
Color + odor	0.1276	0.0951	0.4159	0.0089	0.1484	0.1509	0.1711	0.0175
Color + deposits	0.1292	0.0761	0.3952	0.1999	0.1430	0.1050	0.1343	0.0899

cfu = colony-forming units per gram sample. n.c. = normal counts according to VDLUFA (2017) [51]. Cohen’s Kappa (ĸ) < 0 = no agreement; 0 < ĸ < 0.2: slight agreement; 0.21 < ĸ < 0.4 = fair agreement; 0.41 < ĸ < 0.6 = moderate agreement.

**Table 9:** Association between hygienic deviations and the occurrence of coughing after feeding in different forage types (according to table analysis and chi-squared or exact Fisher test; control group = routine examination with *n* = 55 forage samples, thereof 18 straw and 37 hay samples).

Influencing Variable	Level of Significance (Effect = Pre-Reported Coughing)					
	Roughage		Hay		Straw	
	*n*	*p*-Value	*n*	*p*-Value	*n*	*p*-Value
Detection of storage mites *	135	0.1255	87	0.1123	48	0.9546
Molds **	135	0.8730	87	0.9009	48	0.5201
Yeasts **	115	0.7159	71	0.9445	44	0.1314
Aerobic bacteria **	115	0.8731	74	0.9827	41	0.3124
LPS ***	29	0.2273	16	0.0625 ^1^	13	0.5571 ^1^
Cultivation of *Aspergillus* spp.	135	0.0118	87	0.0216	48	0.9298

* Detected by loupe view. ** Exceedance of normal counts (cfu/g) according to VDLUFA (2017) [51]. *** Exceedance of normal levels (µg/g) according to Kamphues et al. (1986) [50]. ^1^ According to exact Fisher’s test.

**Table 10:** Quantitative determination (log^10^ cfu g^−1^) of *Aspergillus* spp. in forage samples without and with the occurrence of coughing after feeding (*p* = 0.0313).

Preliminary Report	*n*	Counts of *Aspergillus* spp. (log^10^ cfu g^−1^)				
		Mean	s.d.	s.e.	Min	Max
Routine examination	59	1.48	2.08	0.27	0.00	6.08
Coughing	89	2.25	2.17	0.23	0.00	6.74

cfu = colony-forming units per gram sample. s.d. = standard deviation; s.e. = standard error.

## Data Availability

Data supporting the reported results can be found in the archive of examination reports of the Institute for Animal Nutrition, University of Veterinary Medicine Hannover, Foundation, Hannover, Germany.
